# Antibiotic resistance in Sudan: assessing the knowledge and practices of healthcare workers in Khartoum

**DOI:** 10.1093/jacamr/dlae049

**Published:** 2024-04-24

**Authors:** Einas A Osman, Sara A Omer, Rashida M A Elmubarak, Manal Abdelnabi, Safaa Abdelgadir, Dalal G Ahmed, Mohamed H Arbab Nasr, Muna Yousif, Maowia Mukhtar, Leena Al-Hassan

**Affiliations:** College of Applied and Health Sciences, A’Sharqiyah University, Ibra, Oman; Department of Clinical Chemistry, International University of Africa, Khartoum, Sudan; Department of Clinical Microbiology and Infectious Diseases, Soba University Hospital, Khartoum, Sudan; Department of Infection Control, Fedail Hospital, Khartoum, Sudan; Department of Neonatal Intensive Care Unit (NICU), Al-Ribat University Hospital, Khartoum, Sudan; Department of Neonatal Intensive Care Unit (NICU), Al-Ribat University Hospital, Khartoum, Sudan; Paediatric Department, Tathleeth General Hospital, Bisha, Kingdom of Saudi Arabia; Department of Medical Microbiology, Omdurman Ahlia University, Khartoum, Sudan; Department of Microbiology, Countess of Chester Hospital NHS Foundation Trust, Chester CH2 1UL, UK; Bioscience Research Institute, Khartoum, Sudan; Department of Global Health and Infection, Brighton and Sussex Medical School, 3.11 Medical Teaching Building, Falmer, Brighton BN1 9PX, UK

## Abstract

**Background:**

Antibiotic resistance (ABR) is a major public health issue, associated with increased patient morbidity and mortality globally, with significantly higher rates in low- and middle-income countries (LMICs). Assessment of contextual factors, such as information, education, infrastructure and regulations are important for developing local solutions against ABR.

**Objectives:**

To determine the knowledge and practices of healthcare workers (HCWs) towards ABR in hospitals in Sudan.

**Materials and methods:**

A survey was conducted in three different hospitals in Khartoum, Sudan from February to December 2020. HCWs of different specialties and expertise were invited to participate. Data were descriptively analysed using Statistical Package for Social Sciences (SPSS).

**Results:**

ABR was identified as a big challenge by 89% of 345 HCWs who participated. The results show that 79% of doctors don’t rely on the clinical microbiology laboratory (CML) results for antibiotic prescription or clinical decision-making. Sixty percent of HCWs agreed there are infection prevention and control (IPC) guidelines in their hospital, but 74% of them don’t have access to them, and infrequently receive relevant IPC training. Furthermore, HCWs obtain ABR information from other colleagues informally, not through local data or reports.

**Conclusions:**

Despite adequate knowledge of ABR locally, there are significant contextual technical challenges facing HCWs in Sudan, such as availability of policies and accurate data from CMLs. The results indicate a poor link between HCWs and the CMLs for infection management and it is essential to improve communication between the different hospital departments with regard to ABR transmission, and ensure the effectiveness of local IPC policies based on locally available data.

## Introduction

Antibiotic resistance (ABR) is one of the great challenges facing modern medicine globally. The rise in ABR is predicted to be the main reason for mortality by the year 2050, with a predicted toll of 10 million people per year.^1^ It is particularly prevalent in low-and middle-income countries (LMICs), due to lack of regulatory frameworks and guidelines on surveillance and infection, prevention and control (IPC) and unregulated antibiotic usage in the hospitals and the community.^2^ Furthermore, there is evidence of a poor link between clinical decision-making, antibiotic prescriptions in hospitals and the clinical microbiology laboratories (CMLs), in addition to the weak role of IPC units. The poor surveillance and limited laboratory diagnostic capacities consequently lead to increased prevalence of ABR in the hospitals and community.^3^

Healthcare workers (HCWs) play a leading role in solving this problem, as advocates for rational antimicrobial use, stewards of sustainable effectiveness, and IPC interventions.^4,5^ However, due to poor laboratory capacities in LMICs, many HCWs don’t rely on microbiology results for diagnosing and treating infections, and broad-spectrum antibiotics are commonly prescribed indiscriminately.^6^ Additionally, the poor CML capacity leads to inadequate surveillance data, and consequently weak IPC, which is not based on local prevalence of infections. A number of studies worldwide, and in Africa in particular, highlight the lack of coordinated approach to ABR education for HCWs in LMICs.^5^ The WHO’s Global Action Plan for Combatting Antimicrobial resistance (GAP-AMR) emphasizes the need for all countries to include ABR as a core component of HCWs’ education and training.^7^ Due to the HCWs’ role in prescription, management of infections, and practitioners of IPC, they need to have appropriate knowledge and practices in order to reduce ABR rates. Similarly, the capacity and inadequate resources for CMLs in providing accurate and timely results has a big impact on clinical management of infections, prescription practices and compliance with IPC by HCWs. As with numerous research outputs, studies and data, assessment surveys on HCWs’ knowledge, attitude and practices about ABR are predominantly conducted in high-income countries (HICs). However, these results are not necessarily applicable to the situation in LMICs. It is therefore essential that this knowledge and capacity is assessed, in order to develop effective and appropriate interventions and containment of ABR at the local and international level.^8^ It is vital that local context is well understood when designing interventions.^6^

The Sudanese Antimicrobial Resistance Research Group (S-AMR), which was established in 2019 in collaboration with researchers in the UK, aimed to build strong, active and sustainable capacity in AMR-related research by generating knowledge on ABR data and practices in Sudan, including robust epidemiological data, facilitating the link between clinical practice, CMLs, IPC and antibiotic prescribing, thereby establishing a multidisciplinary group with the common aim of reducing and preventing ABR spread.

The aim of this study was to understand the HCWs’ knowledge and practices towards ABR, IPC and antibiotic stewardship in hospitals in Khartoum, Sudan in order to gain insight into the possible interventions that can be targeted to reduce the burden of infections and ABR, and improve IPC measures locally.

## Methods

### Study design

A cross-sectional, questionnaire-based survey was used, containing 25 questions in three sections. The first section was on the HCW’s awareness of ABR, what organism(s) they find in their local hospital, and their sources of information on ABR. The second section contained questions on the hospitals’ policies and guidelines for infection control and antibiotic stewardship, availability of material, frequency of training, and the role of the IPC and pharmacy teams. The last section was targeted for physicians only as it covered questions on antibiotic prescription practices and the link between clinical practice and the clinical microbiology laboratory (CML). The detailed questions of the questionnaire are available in File S1 (available as Supplementary data at *JAC-AMR* Online).

### Study setting

The study was conducted in three large tertiary referral hospitals in Khartoum, Sudan (Soba Hospital, Al-Ribat Hospital and Fedail Hospital). The questionnaire was distributed to HCWs across different disciplines, expertise and experience, from February to December 2020. The study team informed the HCWs of the study aims and objectives and provided an information sheet (Files S1 and S2) prior to the individual HCW’s verbal consent to completing the questionnaire. No personal identifiable information was collected in the questionnaire. Individual HCWs were only allowed to participate once in the study.

### Data collection

The questionnaire was distributed in paper format, and subsequently responses were collated by the local study team in Khartoum, and data transcribed into an electronic data collection sheet (Excel) for further analysis. Questionnaire details are presented in File S2.

### Data analysis

The data were descriptively analysed using the Statistical Package of Social Science (SPSS) software version 26. The mean and standard deviation (SD) were estimated for numerical variables, as well as absolute numbers (*n*) and percentage (%) of the occurrence of items for categorical variables.

### Ethics

The survey was approved by the hospitals’ management and Ethics Board before commencing. Individual written consent was waived as no personal identifiable information was collected.

## Results

**Figure 1.**
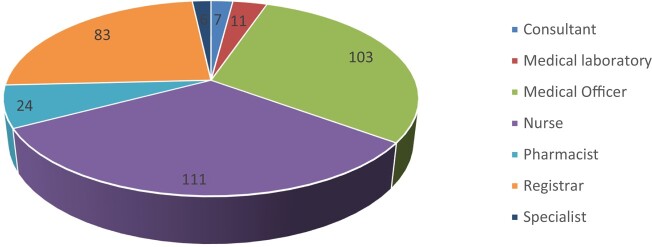
Distribution of HCWs who participated in the study from the three hospitals. Details of specific participants from individual hospitals is provided in Table S1.

A total of 345 HCWs answered the questionnaire in Soba Hospital, Al-Ribat Hospital and Fedail Hospital, with 50, 142 and 153 participants, respectively. Responses were received from different wards and specialties including 7 consultants, 6 specialists, 83 registrars, 103 medical officers, 24 pharmacists, 111 nurses and 11 medical laboratory technicians (Figure 1, Table S1). Respondents’ length of work as HCWs was a maximum of 37 years, with an average of 4.5 years.

### Awareness of ABR in local hospitals

**Figure 2.**
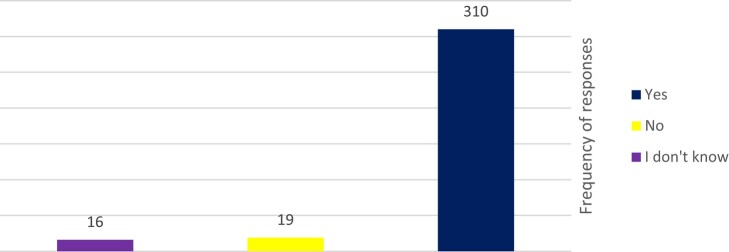
Awareness of HCWs on AMR in their hospitals.

**Figure 3.**
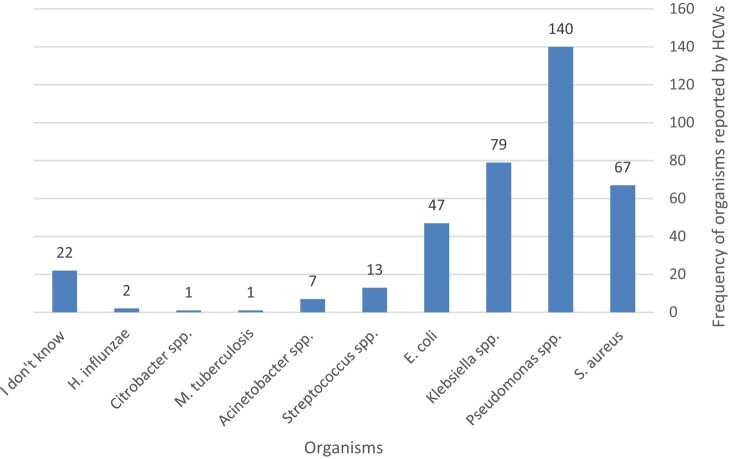
The most problematic antibiotic-resistant bacteria causing nosocomial infections reported by the HCWs. The numbers indicate frequency of the organism reported by individual HCWs. Some HCWs reported more than one bacterial species, hence the larger cumulative number of responses. For *S. aureus*, out of 67 reports, 20 (30%) were MRSA.

A total of 310 participants (89.9%) saw that ABR is a big challenge at their institution (Figure 2), with a total of 140 (40.6%) participants noting that, overall, *Pseudomonas* spp., *Klebsiella* spp. and *Staphylococcus aureus* were the most problematic organisms causing resistant nosocomial infections in their hospitals (Figure 3). On the other hand, 22 participants (6.3% of 345 HCWs) said that they do not know which organism is the most problematic in their hospital.

**Figure 4.**
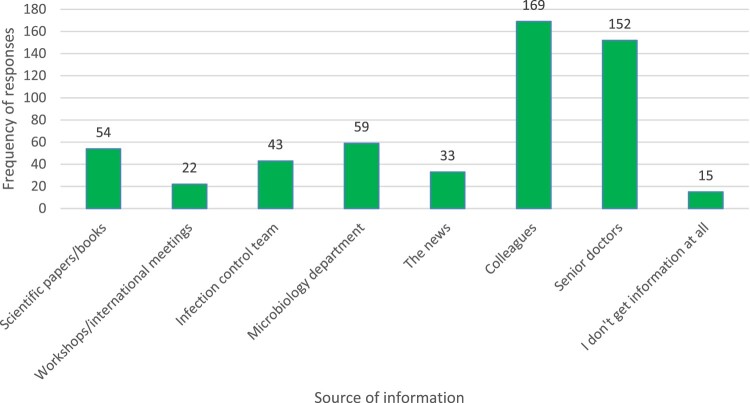
HCWs’ sources of information on AMR. Some HCWs reported multiple sources of information, mostly citing scientific papers/books, together with colleagues and senior staff members as their sources of information.

Variable answers were noted from the HCWs regarding the sources of their information about ABR (Figure 4), the majority of which is received from their colleagues and from the senior doctors: 169 (49%) and 152 (44.1%), respectively. Information from the Microbiology and/or Infection Control Departments was only mentioned by 59 (17%) and 43 (12.4%) of the respondents, while 15 HCWs (4.3%) said they don’t get information at all.

### IPC and antibiotic stewardship in the hospital

Table 1 shows the responses about the presence of and access to IPC policies, training and action in the case of an MDR organism. The majority of participants (*n* = 209; 60.6%) agreed that their hospitals have an IPC policy, 82.2% of whom followed these policies either sometimes (*n* = 142; 68.2%) or always (*n* = 29; 14%). One hundred and seven participants (51.1% of the 209) found it effective in their clinical practice. With regard to the availability of copies of these policies (electronic or paper-based), 74.7% said they don’t have copies, and have only seen/read them in training sessions.

**Table 1.** HCWs’ experience of IPC and antibiotic stewardship guidelines: efficacy, availability and frequency of training

	Frequency	Percent
**Presence of an IPC policy**		
Yes	209	60.6
I don't know	69	20.0
No	67	19.4
If Yes, is it effective?		
I don't know	25	11.9
No	77	36.8
Yes	107	51.1
If Yes, do you follow it?		
No	12	11.2
Rarely	7	6.5
Sometimes	73	68.2
Yes	15	14.0
**Availability of IPC guidelines**		
No	258	74.7
Yes	97	28.1
**Frequency of IPC training**		
Once/year	144	41.7
2–3 times/year	101	29.3
4–5 times/year	24	7
Every 2–3 years	2	0.6
Every month	1	0.3
No training at all	3	0.9
Other	39	11.3
With every new case	30	8.7
**Action taken when a case of multi-drug resistant (MDR) organism is identified in the ward**		
Inform the doctor	119	34.4
Inform infection control	40	11.5
Inform the microbiologist	54	15.6
Inform the senior nurse	33	9.5
No action	99	28.6
**Presence of an antibiotic stewardship policy**		
Yes	187	54.2
No	76	22
I don’t know	82	23.7
**Availability of antibiotic stewardship policy/guidelines**		
No	23	12.3
Rarely	20	10.7
Sometimes	66	35.3
Yes	78	41.7

Twenty percent of participants responded that they didn’t know whether an IPC policy was present and 19.4% said there was no IPC policy in their hospital.

Training in IPC occurs 1–3 times a year, where 26.4% answered that they receive training 2–3 times a year, and 41.7% said once a year. Thirty respondents indicated that they receive IPC training with every new case of an antibiotic-resistant organism detected in the ward. Interestingly, when asked about the action they take if/when an MDR organism is identified in their ward, only 11.5% responded that they would inform the IPC team, 34.4% said they would inform the senior doctor, and 28.6% would take no action.

With regard to antibiotic stewardship, most of the HCWs (*n* = 187; 54.2%) answered yes, the hospital has an antimicrobial stewardship policy, with 77% responses indicating they have a copy of this policy either always or most of the time.

### CMLs and prescription practices

The last section in the questionnaire was for medical doctors only (consultants, specialists or registrars) to gain information about how they manage infections, interaction with the CML, and their prescription practices. In Sudan, only physicians with a medical licence can legally prescribe antibiotics and treat patients.

More than 50% of the doctors said they send specimens for microbiological culture and susceptibility before starting the antibiotic treatment [*n* = 96 (27.8%) most of the time, 84 (24.9%) sometimes], whereas 13 doctors responded that they either don’t or rarely send specimens. However, 158 doctors (80.6%) said the results of the CML are inconsistent with their clinical observations/decisions, and would require them to change the treatments, in comparison to 14 doctors (7.14%) who would continue based on the medical situation only and not the microbiological results. Twenty-four doctors (12.5%) found the microbiology results consistent most of the time with their clinical findings.

When asked about whether the doctors consult with the pharmacy unit on antibiotic doses, 180 doctors (91.3%) said they did, and 13 (6.59%) of them said there is no need for that.

The process of dispensing antibiotics commonly happens through the patient’s family members 43.5% of the time, where 95.5% of physicians answered they mention dose, direction, duration and side effects of using the antibiotic to the patients and their family members, upon prescription and discharge.

As seen in Table 2, diverse classes of antibiotics are prescribed in the hospitals, most frequently being third-generation cephalosporins (29%), followed by a carbapenem (meropenem) at 9%, and amoxicillin/clavulanic acid is less frequently prescribed (7.2%). The doctors described several challenges facing them in the prescription of antibiotics, such as cost and availability of the antibiotics, as well as the increased ABR rates. When asked how they decide whether antibiotics are needed, only one doctor said they rely on microbiological results.

**Table 2.** Frequency of antibiotic prescription for nosocomial infections

Antibiotic class	Antibiotic	*n*	%	Total (%)
Aminopenicillins	Ampicillin			
	Amoxicillin	12	3.5	10.7
	Amoxicillin/clavulanic acid	25	7.2	
Aminoglycoside	Amikacin	3	0.6	
	Gentamicin	6	1.7	
	Tobramycin			
	Streptomycin			
Cephalosporins	First generation: cefalotin/cefazolin	4	12	51.4
	Second generation: cefoxitin/cefuroxime	15	4.3	
	Third generation: cefixime, cefpodoxime/cefotaxime/ceftazidime/ceftriaxone	102	29.6	
	Fourth generation: cefepime	1	0.3	
Flouroquinolones	Ciprofloxacin	21	6.1	7.3
	Norfloxacin	1	0.3	
	Levofloxacin			
Glycopeptide	Vancomycin	6	1.7	
Macrolides	Erythromycin	6	1.8	
	Azithromycin	14	4.1	
Carbapenem	Meropenem	28	9	
Nitroimidazole	Metronidazole	17	4.9	
Combination antibiotics	Trimethoprim/sulfamethoxazole	4	1.2	
β-Lactam antibiotic		8	2.3	
	Colistin	2	0.6	

Total percentage is for the antibiotic group. Some who responded did not specify the specific β-lactam antibiotic prescribed.

## Discussion

This study was conducted to assess the knowledge and practices of HCWs in relation to ABR in the three largest tertiary referral hospitals in Khartoum, Sudan. A total of 345 HCWs participated, with varying degrees of experience, from medical officers to consultants. The majority of responses came from nurses and medical officers (32.2% and 29.9%, respectively). Al-Ribat and Fedail Hospitals contributed to 85.5% of total respondents, while participation was low from Soba University Hospital (14.5%) due to the hospital being semi-locked down during the study period.

ABR is perceived as a big problem by 89.9% of HCWs, and 60.6% agree that their respective hospitals have an IPC policy. However, adherence to IPC policies is quite low at only 14%, and there appears to be no clear policy on reporting of an MDR organism to the IPC departments. Although training takes place 1–3 times a year, and is effective according to HCWs, 68.2% of them only follow local policies and guidelines sometimes. One of the main obstacles was the lack of available copies of these policies and guidelines, hence the lack of adherence. Other studies from LMICs, including Ethiopia, Egypt, Ghana and Uganda, highlight that although theoretical knowledge of ABR is good, context-related barriers in implementation of and adherence to IPC policies were very important to consider.^4,6,8–10^

When asked about the source of information on IPC and ABR, the majority responded that it was through other senior colleagues rather than through the IPC department, indicating the suboptimal link between the IPC department, the CML and the medical staff. In a similar study done in Congo regarding the sources of HCWs’ knowledge of ABR, most respondents said they got the information from their colleagues and the senior doctors, as well as pharmaceutical companies.^11^ Similarly, a study conducted in Egypt indicated that HCWs acquired information on ABR from international guidelines (19.8%), senior colleagues (17%) and pharmaceutical companies (16%).^9^ In Uganda, pharmaceutical companies provided incentives to HCWs that influence prescription practices.^6^ None of the respondents in the current study in Sudan mentioned any role of external pharmaceutical companies impacting their decision-making or practices, which could be due to the political instability and sanctions on Sudan since 1993 preventing big pharmaceutical companies from having a market in Sudan. The data indicate the need to strengthen the role of IPC teams, in providing HCWs with a structured programme of training in IPC and stewardship, and the need to have the policies and guidelines easily available to all staff. In a study conducted by Kheder^12^ in Khartoum, the recommended interventions for combating ABR were (i) educational programmes, and (ii) regular updated ABR rates and antibiograms being available for all physicians. However, working in an overburdened healthcare system comes with additional challenges, such as limited time to attend training sessions even if they were provided, as highlighted by Kagoya *et al*.^6^

More than half of the physicians said they send specimens for microbiological culture and susceptibility testing; however, 35.6% continue treatment based on the clinical situation only and do not rely on the microbiology data. The limited use of microbiological diagnostics in Sudan, plus the reliance on the patient and their families to send the specimen to the microbiology lab and subsequently retrieve the results, certainly impacts the availability of robust and accurate data. The results of this study are consistent with studies conducted in other LMICs (Ethiopia,^8^ Zambia,^13^ Peru^10^ and Egypt^9^), highlighting the poor link between different hospital wards and CMLs, lack of clinical specimens being sent for microbiological culture and susceptibility, and starting antibiotic treatment based on clinical findings, not being confirmed microbiologically (species and antibiogram). In a previous study,^14^ we noted a surge in private laboratory diagnostic services that are replacing the hospital microbiology laboratories. Doctors are increasingly depending on outsourcing microbiological specimens, which results in poor and fragmented hospital data on burden and prevalence of bacteria and ABR.

Inadequate technical and human resources are one of the main barriers to sustainable and successful ABR programmes in LMICs. As presented in a Ugandan study by Kagoya *et al.*,^6^ reliance on the CML was limited due to power challenges, mechanical problems, lack of supplies and technical challenges, which consequently lead HCWs to base their management and antibiotic prescriptions on clinical examinations only, as well as their prior experience with certain types of antibiotics.

As observed in the data from this study, broad-spectrum antibiotics (cephalosporins, third-generation ones in particular) are therefore the most frequently prescribed in Sudan. It is also quite alarming that meropenem is the second-most frequent antibiotic prescribed according to HCWs, indicating high levels of ABR requiring last-line antibiotic treatment. The study by Kheder^12^ states that ceftriaxone and amoxicillin/clavulanic acid were the most commonly prescribed antibiotics, followed by meropenem and vancomycin for resistant infections. When asked about the antibiotic prescription practices, medical practitioners not only consider the patient’s clinical presentation, but also their financial status as the antibiotics are privately available from pharmacies (data not shown). The cost of certain antibiotics (such as carbapenems) is quite high and the patient and their family may be unable to source them. Furthermore, not all antibiotics are readily available in the market, adding another factor that medical practitioners must consider in their prescription practices. Numerous studies have indicated several individual and external factors shaping the prescription practices of physicians in LMICs, such as the personal experience of physicians, the patient demands for antibiotics, and financial incentives by pharmaceutical companies.^8,9^

Most of the HCWs noted that *Staphylococcus* spp. (including MRSA), *Pseudomonas* spp. and *Klebsiella* spp. represented the most problematic resistant organisms causing problems in their wards. Complementary to the questionnaire, the study also included a surveillance study of the burden of Gram-negative bacteria in the hospitals (data not shown), which indicated that *Pseudomonas* spp. and *Klebsiella* spp. are the most common Gram-negative organisms isolated, followed by *Escherichia coli*, thereby confirming the observation of the HCWs. We are unable to confirm *S. aureus* rates; however, the literature suggests high prevalence in Sudanese hospitals.^15^ Similar data were presented in a previous study in Sudan, highlighting that *S. aureus*, *E. coli* and *Pseudomonas* spp. were the most common pathogens encountered.^12^

This questionnaire was part of a larger S-AMR project, which aimed to build strong, active and sustainable capacity in AMR-related research by generating knowledge on AMR data and practices in Sudan, including microbiological and epidemiological data to support strategies to reduce ABR spread. Unfortunately, due to the ongoing conflict in Sudan, we are currently unable to continue the molecular epidemiological investigations of the bacteria collected. The healthcare system has been destroyed by the ongoing conflict.^16^ Evidence shows that conflict has the potential to accelerate and spread AMR locally and globally,^17^ and we are therefore committed to re-establishing the S-AMR group, and continue building the capacity for research, surveillance and supporting intervention-based studies, when the situation allows.

It is important to note that this study took place during the COVID-19 pandemic, where countries and institutions were in lockdown, and healthcare facilities in Sudan and globally were under massive pressures. Despite asking the respondents to answer based on all their years’ experience in healthcare, we cannot rule out any bias in responses due to the situation during the pandemic. One of the main limitations of surveys is that participants may tend to give socially desirable answers. However, we believe that conducting this study, nonetheless, provided insight into the understanding of Sudanese HCWs on ABR, and local practices. Combating ABR requires multidisciplinary collaboration to address rational use of antimicrobials, changes in prescription habits of HCWs, regulation of over the-counter availability of antibiotics, improvements on hand hygiene, and IPC. Furthermore, HCWs need to govern appropriate knowledge, attitudes and practices towards antimicrobial prescription. Information on HCWs’ knowledge and awareness on ABR will permit the development of effective interventions and containment of ABR locally that fit within the global strategies against ABR.^18^

In conclusion, the questionnaire was designed to capture key information on the HCWs’ understanding of ABR and gain insight into local practices. The results reflect a good level of knowledge of ABR in the hospitals in Sudan but suboptimal IPC and stewardship support to HCWs in terms of availability of guidelines and training. The study sheds light on the practices related to antibiotic prescribing in healthcare settings in Sudan, where the physicians rarely rely on microbiological culture and susceptibility results, but also must consider the patients’ financial and social status due to the antibiotics being purchased privately.

We believe that robust epidemiological surveillance combined with context-driven interventions are urgently needed, with tailored actions that address the specific challenges highlighted in this study. ABR is a One Health issue, and therefore needs a coordinated multifaceted response.

## Supplementary Material

dlae049_Supplementary_Data

## Supplementary data

Files S1 and S2 and Table S1 are available as Supplementary data at *JAC-AMR* Online.
