# Supplementary material for: Antibiotic resistance in Sudan: assessing the knowledge and practices of healthcare workers in Khartoum
Source: JAC Antimicrob Resist. 2024 Apr 24;6(2):dlae049. doi: 10.1093/jacamr/dlae049 (PMC11040271; doi:10.1093/jacamr/dlae049)
Supplement: dlae049_Supplementary_Data [file dlae049_supplementary_data.docx]

Supplementary Table 1 (Table S1): Distribution of HCWs from individual hospitals

|  | | Hospital | | | Total |
| --- | --- | --- | --- | --- | --- |
|  |  | Soba University Hospital | Alrebat Hospital | Fedial Hospital |  |
| Profession | Consultant* | 0 | 3 | 4 | 7 |
|  | Medical laboratory | 0 | 0 | 10 | 10 |
|  | Medical Officer* | 10 | 55 | 38 | 103 |
|  | Nurse | 19 | 47 | 45 | 111 |
|  | Pharmacist | 8 | 0 | 16 | 24 |
|  | Registrar* | 12 | 35 | 36 | 83 |
|  | Specialist* | 1 | 2 | 3 | 6 |
| Total | | 50 | 142 | 152 | 345 |

Numbers indicate the number of participants who answered the survey.

*The distinction between the physicians is in years of experience as follows: Medical officer: finished internship work as house officer for one year in 4 specialities and finished registration exam; Registrar: started specialist training after registration exam for 4 years; Specialist: Finished training and finished exam; Consultant: after 5 years of being specialist.

**Supplementary file 1:**

**Survey on the understanding and practices related to antimicrobial resistance in hospitals in Khartoum, Sudan.**

**Thank you for taking the time to do this survey.**

**This is a brief questionnaire to assess your understanding and practices, as a healthcare worker, of the burden of Antimicrobial Resistance (AMR) in your institution, and how we can create efficient locally-relevant practices and policies in infection prevention and control, and antimicrobial stewardship.**

**Your answers will be accessible by the study team at the Bioscience Research Institute in Khartoum, as well as the collaborator, Dr Leena Al-Hassan at the University of Sussex. The data generated from the study will be used for research publications, as well as feedback to the hospital management.**

**By answering this survey, you are consenting to participation, and the information you provide can be used for future research publications, and intervention-based research projects. If you do not wish to participate in the study, please hand the sheet back to …………………………………………… (Name to be filled in by the respective study team member at the hospital).**

**Please answer to the best of your knowledge and if you have any questions please do ask the study team in your hospital …………………………………………… (Name to be filled in by respective study team member at hospital), or Dr Leena Al-Hassan at** [**l-al-hassan@bsms.ac.uk**](mailto:l-al-hassan@bsms.ac.uk)**.**

**Please do not add any personal identifiable information to the survey.**

**­­­­**

­

**Profession:**

Nurse ☐ House officer ☐ Medical officer ☐ Registrar ☐ Specialist ☐ Consultant ☐ Pharmacist ☐

**Years of experience:**

**1) Awareness of Antimicrobial Resistance (AMR)**

1.1 Do you see AMR as a big problem at your institution?

☐Yes

☐No

☐I don’t know

1.2 Which organisms pose the greatest problem in your ward?

1.3 How do you get information about the problem of AMR? (Tick as many as you want)

☐Colleagues

☐Senior doctors

☐The microbiology department,

☐The infection control nurse,

☐ The news

☐Papers/Books

☐Workshops/international meetings

☐I don’t get information at all

**2) Infection control policies**

2.1 Does the hospital have infection control policies?

☐Yes – go to section 2.2

☐No – go to section 3

☐I don’t know

2.2 If yes, do you find it effective?

☐ Yes

☐ No

☐ I don’t know

2.3 If yes, do you follow it?

☐Yes, all the time,

☐ Sometimes,

☐ Rarely,

☐ No

- 1. Do you have a copy of these policies?

☐Yes

☐ No

2.5 How often do you have training on infection prevention and control?

☐Every month

☐Every 3 months.

☐ Every 6 months.

☐No training at all.

☐Others how many times…………………………

2.6 What actions do you take when you have a case of multi-drug resistant (MDR) organism in your ward?

☐ Inform the doctor,

☐ Inform the senior nurse,

☐ Inform the infection control nurse,

☐ Inform the microbiologist,

☐ Nothing.

- 1. Do you have cases of Pan-drug resistance (PDR) at the hospital? **Resistant to all antibiotics**

☐ Yes

☐ No

2.8 Do you have information about which wards show the highest infection rates with MDR?

**3) Stewardship: systems and processes for effective antimicrobial medicine use**

3.1 Are you aware of an antimicrobial stewardship policy at the hospital?

☐ No

☐ Yes

- 1. If yes, do you have a copy?

☐ No

☐ Yes

**For Doctors only**

How often do you send specimens for culture and sensitivity before starting the antibiotic treatment?

☐ Most of the time,

☐ Sometimes,

☐ Rarely,

☐ Not at all

Do you find the laboratory report consistent with your clinical findings?

☐ Most of the time

☐ Sometimes

☐ Rarely

☐ Never

Would you change the antibiotic prescription after culture results or continue based on clinical situation?

☐ Change it

☐ Continued based on clinical situation

☐ Other (specify)

How often do you consult a microbiologist on managing a serious infection?

☐ All the time

☐ Sometimes

☐ I do not consult them

☐ I do not know where the microbiologist is based

What is the process of dispensing antibiotics from the pharmacy?

Do you consult the pharmacy on antibiotic doses?

☐Yes

☐ No

☐ Sometimes

What types of antibiotics are most commonly prescribed? Why?

What are the problems/challenges/that facing you in prescribing of antibiotics?

Do you mention dose, direction, duration, side effects (or AMR) of using antibiotic to the

patients?

Which group of antibiotics are prescribed most?

☐ First line/generation,

☐ Second line/generation or

☐ Third line/generation

Why?

How do you decide whether to prescribe antibiotic for a particular patient?

**Supplementary file 2:**

**Sudanese AMR (S-AMR) Research Group: Proposed Research Plan Jan-July 2019**

The main aim of the S-AMR research group/network is to generate knowledge on AMR-related data and practices in Sudan, focusing on antibiotic resistance in healthcare settings.

We aim to create a link between clinical practice, clinical microbiology, infection prevention and control (IPC) policies, and antibiotic prescribing, thereby establishing a multidisciplinary group with the common aim of reducing and preventing AMR spread.

There is furthermore an urgent need for an **active** & **sustainable** collaborative link between clinical microbiology in hospitals and academic research centres in Sudan, hence we are proposing the establishment of a collaboration between Soba University Hospital Microbiology Department, Al Rabat Hospital Microbiology Department & The BioScience Research Institute based at Ibn Sina University. This link will facilitate the molecular microbiology work of the project locally in Sudan, which will be supported by the Department of Global Health and Infection at Brighton and Sussex Medical School.

What knowledge do we (locally) want to know?

The aim of the project is to build capacity in AMR research in Sudan by creating a link between Clinical Microbiology, IPC and prescribing practices to generate knowledge AMR locally in Sudan.

Microbiology

Bacterial data collection – Focus on problematic Gram-Negatives: *Klebsiella & Acinetobacter*

Gram-Negative bacteria will be collected from the microbiology laboratory (by a clinical microbiologist and a laboratory technician), for which epidemiological data will be collected from the patient records. All the data will be anonymised. The epidemiological data (CRF attached) will give us insight into the clinical manifestations of the causative organism, and also enable us to find common risk factors with acquisition and dissemination in the hospital.

Unidentified isolates will undergo genomic identification by 16s-23s rRNA typing in collaboration with The Bioscience Research Institute at Ibn Sina University.

Antimicrobial susceptibility data will be collected from the routine clinical microbiology work which is performed by disk diffusion. Some strains (e.g. MDR and part of the ESKAPE pathogen list) will furthermore undergo MIC testing by broth microdilution method. This will be decided on a case-by-case basis.

At the end of the project we aim to:

1. Have established a biobank for microorganisms in Sudan that will facilitate future research
2. Choose a subset of isolates to undergo whole genome sequencing (WGS) to determine molecular epidemiology and characterise resistance mechanisms.

Antibiotic Prescription, Stewardship, and Infection Control

A survey will be distributed to healthcare workers in order to assess their understanding of the burden of AMR, and how this is affecting antimicrobial prescribing in the hospital. (Questionnaire/survey attached).

We also aim to review any infection prevention and control (IPC) guidelines and/or policies present at the hospital, and how they are adapted in the clinical setting.

For efficient execution of the project we need to build a team of good researchers. The aim is to recruit bright and motivated junior researcher(s) (doctors/pharmacists/laboratory technicians) who have an interest in AMR research and who are willing to commit part of their time to the research projects. This will be a funded post for the duration of the study (February 2020-June 2020), and they will be included in the authorship of any research outputs/publication. This will help in building local capacity and engaging early-career researchers (ECRs)

The project is running for from January-July 2020 will be a startup for future work in S-AMR. We want to build capacity for AMR research in Sudan, and create a sustainable research network of committed scientists.

We will work towards using the data produced to improve IPC, establish antimicrobial stewardship programmes, thereby creating a locally-relevant intervention in the future. Furthermore, we want to emphasize the importance of national and international collaboration and the need for multidisciplinarity in AMR research.

All the project outcomes will be published in academic journals, shared with hospital management to discuss how we can sustain and grow the research in AMR, and what areas need further focus.
